# Tobacco consumption in the Kingdom of Saudi Arabia, 2013: findings from a national survey

**DOI:** 10.1186/s12889-015-1902-3

**Published:** 2015-07-05

**Authors:** Maziar Moradi-Lakeh, Charbel El Bcheraoui, Marwa Tuffaha, Farah Daoud, Mohammad Al Saeedi, Mohammed Basulaiman, Ziad A. Memish, Mohammad A. AlMazroa, Abdullah A. Al Rabeeah, Ali H. Mokdad

**Affiliations:** Institute for Health Metrics and Evaluation, University of Washington, 2301 Fifth Ave., Suite 600, Seattle, WA 98121 USA; Ministry of Health of the Kingdom of Saudi Arabia, Assadah, Al Murabba, Riyadh, 12613 Saudi Arabia

**Keywords:** Tobacco, Smoking, Shisha, Saudi Arabia

## Abstract

**Background:**

Tobacco consumption is a major risk factor for morbidity and mortality. The Saudi Ministry of Health started a national tobacco control program in 2002 with increased and intensified efforts after joining the World Health Organization Framework Convention for Tobacco Control in 2005.

**Methods:**

In order to assess the status of tobacco consumption in the Kingdom of Saudi Arabia (KSA), we conducted a survey on 10735 individuals aged 15 years or older (5253 men and 5482 women) which was performed between April and June 2013. The Saudi Health Interview Survey had a multistage sampling and was nationally representative. Data were collected through face-to-face interviews. The survey included questions on socio-demographic characteristics, tobacco consumption, diet, physical activity, health care utilization, different health-related behaviors, and self-reported chronic conditions.

**Results:**

Overall prevalence of current smoking was 12.2 % and males were more likely to smoke than females (21.5 % vs. 1.1 %). Mean age of smoking initiation was 19.1 years (±6.5 years) with 8.9 % of ever smokers starting before the age of 15 years. Daily shisha smoking was reported by 4.3 % of the population (7.3 % of men and 1.3 % of women). Around 1.4 % of population (2.6 % of men and 0.1 % of women) were daily smokers of cigarette/cigar and shisha. Receiving advice for quitting smoking by health care professionals during the last 12 months was reported by 53.2 % (95 % confidence interval [CI]: 49.8–56.5) of ever smokers. Among ever smokers, 51.3 % of individuals reportedly attempted to quit smoking during the last 12 months. Of those, 25.3 % were successful by the time of the survey. Around 23.3 % of the entire population, 32.3 % of men and 13.5 % of women, were exposed to secondhand smoke for at least one day during the past 7 days at home, work, or school.

**Conclusions:**

Although the indicators of tobacco consumption in KSA are better than most of the countries of the Middle East region and high-income countries, there are many potential areas for improvement. Our findings call for the development and implementation of programs to prevent smoking initiation and encourage quitting. To achieve its health goals, KSA may consider increasing taxation on tobacco products as well as other measures.

## Background

Tobacco consumption is a major risk factor for morbidity and mortality.[1] The Saudi Ministry of Health started a national tobacco control program in 2002 with increased and intensified efforts after joining the World Health Organization (WHO) Framework Convention for Tobacco Control (FCTC) in 2005. [2] The Kingdom of Saudi Arabia (KSA) has banned smoking in most public places, including health care facilities, all educational settings, government facilities, restaurants and cafés, and the public transport system; national law requires fines for smoking, which are levied on the smoker. However, there are no dedicated funds for enforcement or specific mechanisms for citizen complaints and investigations. The law mandates health warnings on tobacco packages, which include a photograph or graphic. Selling tobacco products to minors is prohibited. The price of a pack of 20 cigarettes in KSA is around US $ 1.10 for the cheapest brand and around US$ 2.70 for Marlboro (the most popular brand); these prices include 22 % tax (basically equivalent to import duty). In KSA, treatments for smoking cessation, such as nicotine replacement therapy, bupropion, and varenicline are legally available with a prescription [3, 4].

Based on article 20.2 of the FCTC, countries shall establish programs for surveillance of the magnitude, patterns, determinants, and consequences of tobacco consumption and exposure to tobacco smoke.[5] In KSA, current data on tobacco consumption are lacking. Some studies reported that tobacco consumption has increased in the Arab World and KSA. [6, 7] However, there are few studies from KSA that reported smoking status from a population-based sample. [8–10] The last nationally representative data on tobacco consumption in KSA are from the 2005 STEPwise approach survey, which reported a 22.2 % prevalence of current smoking in men and 1.4 % in women [11]. Other studies reported smoking status amongst specific populations. For instance, Abdel Rahim reported a 23.5 % prevalence of current smoking among attendees of primary health care facilities in Jizan in 2012. [12] Another study reported that among military personnel, 35 % were current smokers in 2009 to 2011. [13] In order to assess the current status of tobacco consumption (ever smoking, current smoking, exposure to secondhand smoke, shisha smoking, smokeless tobacco consumption, and quitting behavior) in KSA, we conducted a large national health survey in 2013.

## Methods

The Saudi Health Interview Survey was a cross-sectional national multistage survey of individuals aged 15 years or older performed between April and June 2013. KSA was divided into 13 regions. Each region was divided into subregions and blocks. All regions were included and a probability proportional to size was used to randomly select subregions and blocks. Households were randomly selected from each block. A roster of household members was conducted and an adult aged 15 or older was randomly selected to be surveyed. If the randomly selected adult was not present, our surveyors made an appointment to return, and a total of three visits were made before the household was considered as a nonresponse. The sample was nationally representative for all Saudi nationals 15 years or older. [14–16]

The Saudi Ministry of Health and its Institutional Review Board (IRB) approved the study protocol. The University of Washington IRB deemed the study as IRB exempt, since the Institute for Health Metrics and Evaluation received de-identified data for this analysis. All respondents consented and agreed to participate in the study. If the randomly selected respondent was between the ages of 15–17 years old, then the parent(s) or legal guardian of that individual consented as well.

The survey included questions on socio-demographic characteristics, tobacco consumption, diet, physical activity, health care utilization, and self-reported chronic conditions.

Smoking status was assessed using 15 questions. Ever smoking was assessed by asking respondents about smoking any tobacco products, such as cigarettes, cigars or pipes, or Shisha. To assess current smoking, respondents were asked for current use and current daily smoking of tobacco products. Smokers were also asked about the amount of their tobacco use.

The current or previous smokers were asked for their ages when they first started smoking daily.

The ever smokers were asked for quitting attempts and receipt of advice to stop smoking during the past 12 months.

Smokeless tobacco status was assessed by asking for ever and current use of smokeless tobacco products, such as snuff, chewing tobacco, Swaika, or Medwakh. To assess exposure to secondhand smoke, respondents were asked on how many of the past 7 days someone smoked in their home when they were present at home, and similar questions were asked for their workplace or school.

We used measured weight and height to calculate body mass index (BMI) as weight (kg)/height (m2). Participants were classified into four groups: 1) underweight for a BMI less than 18.5; 2) normal weight for a BMI within 18.5–25.0; 3) overweight for a BMI within 25.0–30.0; or 4) obese if their BMI was greater than or equal to 30. We used the International Physical Activity questionnaire to classify respondents into four groups of physical activity [17]: (1) met vigorous physical activity, (2) met moderate physical activity, (3) insufficient physical activity to meet vigorous or moderate levels, and (4) no physical activity. For analytic purposes, we used no (minimal) physical activity against all other groups. We computed the servings of fruits and vegetables consumed per day from the detailed dietary questionnaire as the sum of the average daily servings.

The respondents were asked to rate their health as excellent, very good, good, fair, or poor and compare it with 12 months ago as better, worse, or about the same.

To assess diagnosed hypertension, diabetes, and hypercholesterolemia status, respondents were asked four separate questions to understand whether they have ever been told by a doctor, nurse, or other health professional that had pre-diabetes mellitus (otherwise known as pre-diabetes, borderline diabetes, impaired fasting glucose, impaired glucose tolerance, or impaired sugar tolerance), diabetes mellitus, hypercholesterolemia, or hypertension. Women diagnosed with diabetes or hypertension during pregnancy were not counted as having these conditions. Similarly, the same type of questions was used to determine previous diagnosis of stroke, myocardial infarction, atrial fibrillation, cardiac arrest, congestive heart failure, chronic obstructive pulmonary disease, asthma, renal failure, and cancer. We considered a person to be diagnosed with a chronic condition if they reported being diagnosed with any of these conditions.

We used a backward elimination multivariate unconditional logistic regression model to measure association between the outcome variables of current smoking, daily shisha consumption, heavy smoking (more than 16 cigarettes per day), and successful attempts to quit smoking and covariates, including sex, age, marital status, education, diet, physical activity, self-rated health, and reported chronic conditions. Of the 10,735 completed interviews, all had age and sex data, but we excluded 29 observations for missing smoking status, 122 observations for missing daily shisha consumption, 398 observations for missing obesity values, 266 for missing self-reported hypercholesterolemia status, 115 for missing self-reported diabetes status, 32 for missing self-reported other chronic conditions, 212 for missing fruit and vegetables consumption, 33 for missing marital status, and 20 for missing educational level. In total, 10,293 observations were used in our regression analyses.

Data were weighted to account for the probability of selection and age and sex poststratification based on census data for age and sex distribution of the Saudi population. We used Stata 13.1 for windows (StataCorp LP, TX, USA) for the analyses and to account for the complex sampling design.

## Results

A total of 12,000 households were originally contacted, and 10,735 participants completed the SHIS questionnaire (response rate: 89.4 %). Characteristics of respondents who completed the questionnaire are presented in Table 1.

**Table 1:** Socio-demographic characteristics of the Saudi Health Interview Survey participants, Kingdom of Saudi Arabia, 2013

Socio-demographic characteristics	Categories	Number	Weighted %	SE
Sex	Male	5253	50.6 %	0.7
	Female	5482	49.4 %	0.7
Age (years)	15–24	2382	40.3 %	0.7
	25–34	2757	21.5 %	0.5
	35–44	2339	15.2 %	0.4
	45–54	1520	12.4 %	0.4
	55–64	862	6.5 %	0.3
	65 or older	875	4.2 %	0.2
Marital status	Never married	2829	45.7 %	0.7
	Currently married	6976	49.2 %	0.7
	Separated, divorced, or widowed	929	5.1 %	0.2
Education	Primary school or less	3286	26.3 %	0.6
	Elementary or high school	4780	51.9 %	0.7
	College or higher education	2649	21.8 %	0.5

SE: standard error

Among Saudis aged 15 years or older, 16.0 % ever smoked tobacco, and 12.2 % were current tobacco smokers. The mean age of ever daily smoking initiation was 18.8 years (standard error [SE] = 0.22) for males and 19.7 years (SE = 1.34) for females. Smoking status varied by sex, age group, educational group, and marital status (Table 2). Approximately 1.4 % (SE = 0.2) of the population were daily smokers of both cigarette/cigar and shisha; this percentage was 2.6 % in men and 0.1 % in women. While prevalence of current daily smoking of all tobacco products in men is around 19.6 times that of women, the male-to-female ratio of prevalence of daily shisha smoking is 5.6.

**Table 2:** Smoking status in Saudi Adult by socio-demographic and selected health conditions, 2013

Characteristics	Categories	Former smokers			Current, non-daily smoking			Current, daily smoking			Daily shisha consumption		
		N	%	SE	N	%	SE	N	%	SE	N	%	SE
Sex	Both (Total)	458	3.8 %	0.2	79	0.8 %	0.1	1277	11.4 %	0.4	480	4.3 %	0.3
	Male	429	6.9 %	0.4	60	1.2 %	0.2	1200	21.5 %	0.8	406	7.3 %	0.5
	Female	29	0.6 %	0.0	19	0.4 %	0.0	77	1.1 %	0.0	74	1.3 %	0.2
Age (years)	15–24	50	1.8 %	0.3	17	0.7 %	0.3	196	7.9 %	0.7	75	3.0 %	0.5
	25–34	89	4.1 %	0.5	25	1.0 %	0.2	385	15.0 %	1.0	128	5.2 %	0.6
	35–44	111	4.3 %	0.5	20	0.8 %	0.2	335	13.8 %	0.9	120	4.5 %	0.5
	45–54	68	5.1 %	0.8	9	0.5 %	0.2	212	14.0 %	1.2	77	5.1 %	0.7
	55–64	70	7.8 %	1.8	5	0.9 %	0.3	104	14.7 %	1.7	52	7.4 %	1.4
	65+	70	9.3 %	1.4	3	0.4 %	0.3	45	6.1 %	1.2	22	4.6 %	1.2
Marital status	Never married	77	2.2 %	0.3	18	0.7 %	0.2	317	9.7 %	0.7	100	3.2 %	0.4
	Currently married	363	5.5 %	0.4	58	0.9 %	0.1	889	13.5 %	0.6	352	5.4 %	0.4
	Separated, divorced, or widowed	18	1.6 %	0.5	3	0.5 %	0.4	71	7.4 %	1.1	28	3.0 %	0.7
Education	Primary school or less	141	3.6 %	0.4	10	0.3 %	0.1	256	10.0 %	0.8	99	3.5 %	0.5
	Elementary or high school	210	3.8 %	0.4	45	1.0 %	0.2	669	12.1 %	0.6	231	4.4 %	0.4
	College or higher education	106	3.9 %	0.5	24	0.9 %	0.2	350	11.7 %	0.8	149	5.0 %	0.5
Body Mass Index (Kg/m^2^)	<25	93	5.5 %	0.7	13	0.8 %	0.3	297	20.7 %	1.4	84	6.0 %	0.8
	25–29.9	128	5.9 %	0.6	13	0.5 %	0.2	323	13.6 %	0.9	127	5.0 %	0.5
	30+	147	4.8 %	0.5	25	1.0 %	0.2	292	9.9 %	0.8	133	4.5 %	0.5
Minimal physical activity	No	236	3.8 %	0.3	38	0.7 %	0.2	749	12.5 %	0.6	254	4.5 %	0.4
	Yes	68	2.4 %	0.4	22	0.6 %	0.1	255	8.2 %	0.7	114	3.3 %	0.4
Daily servings of fruits/vegetables	Less than 5	436	3.9 %	0.2	66	0.6 %	0.1	1169	11.4 %	0.5	442	4.3 %	0.3
	5 or more	22	2.6 %	0.9	13	2.3 %	1.0	108	12.3 %	1.5	38	4.3 %	0.9
History of diagnosis with hypercholesterolemia	No	367	3.4 %	0.2	69	0.7 %	0.1	1142	11.2 %	0.4	414	4.1 %	0.3
	Yes	67	8.3 %	1.3	6	1.0 %	0.5	107	14.8 %	1.8	48	6.1 %	1.3
History of diagnosis with hypertension	No	383	3.4 %	0.2	67	0.7 %	0.1	1159	11.4 %	0.5	423	4.2 %	0.3
	Yes	69	7.6 %	1.2	9	1.2 %	0.5	106	11.6 %	1.5	47	5.3 %	1.0
History of diagnosis with diabetes	No	429	3.7 %	0.2	76	0.8 %	0.1	1214	11.1 %	0.4	447	4.1 %	0.3
	Yes	12	5.0 %	1.8	0	0.0 %	0.0	33	23.7 %	4.2	14	8.7 %	2.8
History of diagnosis with a chronic condition	No	318	3.2 %	0.2	67	0.8 %	0.1	1079	11.1 %	0.5	383	4.0 %	0.3
	Yes	140	7.9 %	0.8	12	0.9 %	0.3	198	13.5 %	1.2	97	6.2 %	0.8

N: number, %: percentage, SE: standard error

The likelihood of being a current smoker increased with age (adjusted odds ratio [AOR] = 1.12; 95 % confidence interval [CI]: 1.00–1.25), among the married (AOR = 1.70; 95 % CI: 1.21–2.40), among the separated, divorced, or widowed (AOR = 3.55; 95 % CI: 2.02–6.24), and among individuals diagnosed with pre-diabetes (AOR = 2.42; 95 % CI: 1.36–4.32) compared to single individuals and those with no history of pre-diabetes diagnosis; however, the likelihood of being a current smoker decreased among women (AOR = 0.04; 95 % CI: 0.03 – 0.06) compared to men (Table 3).

**Table 3:** Association of socio-demographic characteristics and health risks with current smoking, daily shisha consumption, and heavy smoking (more than 16 cigarettes per day) in logistic regression backward elimination models in Saudi Adults, 2013

Characteristics	Current smoking				Daily shisha consumption				Heavy smoking		
	OR	95 % CI			OR	95 % CI			OR	95 % CI	
Age (decades)	1.12	1.00	1.26		1.09	0.92	1.29		1.24	1.06	1.45
Sex:											
Male	1.00			1.00					1.00		
Female	0.04	0.03	0.06	0.13		0.09	0.20		0.01	0.00	0.02
Education:											
Primary school or less	1.00			1.00				1.00			
Elementary/high school completed	NA			1.33		0.83	2.13	0.77		0.51	1.15
College degree or higher education	0.89	0.71	1.11	1.32		0.81	2.15	0.59		0.38	0.93
Marriage:											
Never married	1.00			1.00				1.00			
Currently married	1.72	1.21	2.43	1.73		1.07	2.77	1.45		0.87	2.42
Separated, divorced, or widowed	3.58	2.03	6.30	2.37		1.20	4.70	2.60		1.09	6.23
Body mass index (kg/m^2^)											
< 25	1.00			1.00				1.00			
25─30	0.81	0.63	1.04	NA				0.82		0.61	1.10
30+	0.89	0.68	1.15	NA				NA			
Minimal physical activity											
No	1.00			1.00				1.00			
Yes	NA			1.24		0.91	1.69	1.15		0.83	1.58
Daily servings of fruits/vegetables											
Less than 5	1.00			1.00				1.00			
5 or more	1.19	0.84	1.69	NA				1.27		0.80	2.02
History of diagnosis with hypercholesterolemia											
No	1.00			1.00				1.00			
Yes	0.75	0.52	1.10	NA				NA			
History of diagnosis with pre-diabetes											
No	1.00			1.00				1.00			
Yes	2.42	1.36	4.32	2.63		1.30	5.33	1.81		0.86	3.80
History of diagnosis with hypertension											
No	1.00			1.00				1.00			
Yes	NA			1.22		0.73	2.04	NA			
History of diagnosis with a chronic condition											
No	1.00			1.00				1.00			
Yes	NA			NA				1.18		0.80	1.74
Constant	0.16	0.12	0.22	0.04		0.02	0.08	0.06		0.04	0.10

Note: OR: odds ratio; CI: confidence interval; NA = not applicable as category has been removed by the model; variables and subcategories with *P* > 0.5 were removed from the model

Among Saudis aged 15 years or older, 4.3 % smoke shisha on a daily basis (Table 2). This consumption pattern is more prevalent among the married (AOR = 1.73; 95 % CI: 1.04–2.77), the separated, divorced, or widowed (AOR = 2.37; 95 % CI: 1.20–4.70), and individuals diagnosed with pre-diabetes (AOR = 2.63; 95 % CI: 1.30–5.33) compared to single individuals and those with no history of pre-diabetes diagnosis, but daily smoking of shisha was less prevalent among women (AOR = 0.13; 95 % CI: 0.09–0.20) compared to men (Table 3).

Among current smokers, 74.1 % smoke cigarettes. The average number of cigarettes smoked per day was 15.1 with 39.4 % being heavy smokers (more than 16 cigarettes per day among current smokers). Older individuals (AOR = 1.23; 95 % CI: 1.05–1.44) and those who are separated, divorced, or widowed (AOR = 2.60; 95 % CI: 1.09–6.17) were more likely to be heavy smokers compared to younger and never married individuals. Women (AOR = 0.01; 95 % CI: 0.00–0.02) and individuals with a college degree or higher (AOR = 0.60; 95 % CI: 0.38–0.94) were less likely to be heavy smokers compared to men and individuals with primary schooling or less (Table 3).

**Fig. 1:**
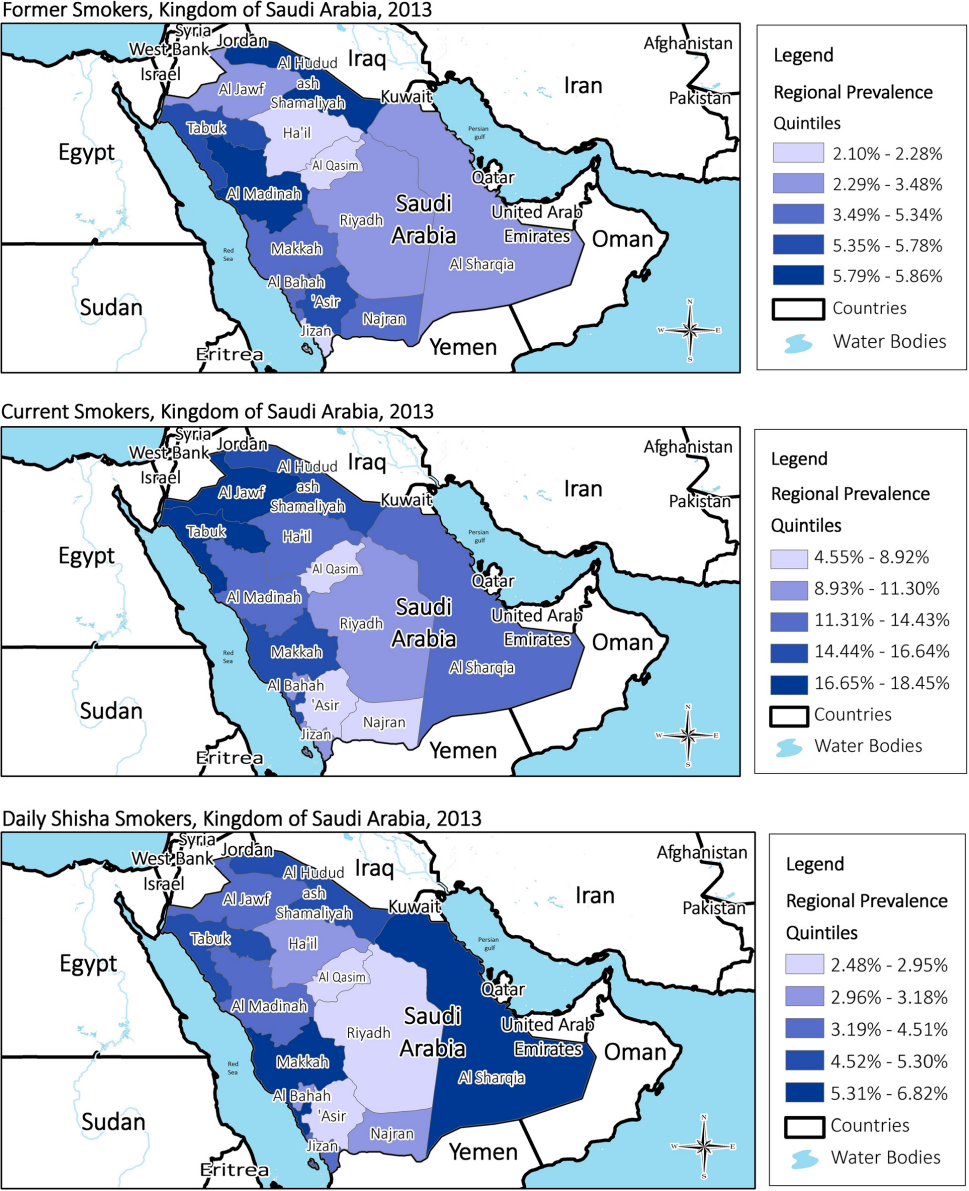
Status of current smoking, daily shisha consumption, and heavy smoking (more than 16 cigarettes per day) in the Kingdom of Saudi Arabia, 2013

There were wide variations in tobacco consumption measures by different regions of KSA (Fig. 1). The highest prevalence of current smoking was observed in Tabuk, Al-Jawf, and Al Hudud Ash-Shamaliyah, at 18.5, 18.3, and 16.6 %, respectively, while the lowest was in’Asir and Najran (4.6 and 4.8 %, respectively). The highest and lowest prevalence of heavy smoking was observed in Tabuk (11.7 %) and Najran (1.2 %), respectively.

Among ever smokers, 160 individuals (9.0 %) had started smoking before the age of 15.

Around 51.3 % (904 individuals) of ever smokers attempted quitting during the previous 12 months; 25.3 % of these attempts were successful by the time of the survey. Among former smokers, 255 (58.7 %) reported that they had quit smoking during the past 12 months, and 163 (41.3 %) had at least 1 year of abstinence. Moreover, 49.2 % (95 % CI: 45.4–53.0) of current smokers reported that they had unsuccessfully tried to quit smoking during the last 12 months. Older age (AOR = 1.29; 95 % CI: 1.08–1.55) and history of diagnosis with a chronic condition (AOR = 2.14; 95 % CI: 1.25–3.68) were positively associated with successful attempts to quit smoking, while being separated, divorced, or widowed (AOR = 0.07; 95 % CI: 0.02–0.23), increased number of cigarettes smoked per day (AOR = 0.94; 95 % CI: 0.91–0.96), and exposure to secondhand smoke (AOR = 0.60; 95 % CI: 0.40–0.90) were negatively associated with such attempts (Table 4).

**Table 4:** Multivariate logistic regression for socio-demographic and other factors associated with successfully quitting smoking, Kingdom of Saudi Arabia, 2013

Characteristics	Odds ratio	95 % CI	
Age (decades)	1.29	1.08	1.55
Education:			
Primary school or less	1.00		
Elementary/high school completed	NA		
College degree or higher education	1.27	0.83	1.93
Marriage:			
Never married	1.00		
Currently married	NA		
Separated, divorced, or widowed	0.07	0.02	0.23
Minimal physical activity			
No	1.00		
Yes	0.71	0.41	1.20
Daily servings of fruits/vegetables			
Less than 5	1.00		
5 or more	0.28	0.13	0.60
Cigarettes per day	0.94	0.91	0.96
Exposure to secondhand smoke			
No	1.00		
Yes	0.60	0.40	0.90
History of diagnosis with hypercholesterolemia			
No	1.00		
Yes	1.62	0.72	3.68
History of diagnosis with pre-diabetes			
No	1.00		
Yes	0.19	0.04	0.82
History of diagnosis with a chronic condition			
No	1.00		
Yes	2.14	1.25	3.68
Constant	0.27	0.13	0.55

NA: not applicable as category has been removed by the model, CI: confidence interval

Sex, body mass index, and “history of diagnosis with hypertension” were removed from the model during backward elimination

Among former smokers who quit during the last 12 months, 35.4 % (95 % CI: 27.6–44.1) reported better general health compared to the previous year; this percentage was 31.1 % (95 % CI: 22.1–41.7) in former smokers with at least 1 year of abstinence, 25.8 % (95 % CI: 21.2–31.0) in current smokers with an unsuccessful quit attempt in the last 12 months, and 23.5 % (95 % CI: 19.4–28.1) in current smokers that had not tried quitting.

In addition, 23.3 % (95%CI: 22.1–24.6) of the entire population, 32.3 % (95 % CI: 30.4–34.3) of men and 13.5 % (95 % CI: 12.1–15.0) of women, were exposed to secondhand smoke for at least one day during the past 7 days at home, work, or school. The ratio of secondhand smoke exposure in males to females was 1.6 for home and 6.4 for work or school. The percentage of work or school exposure to secondhand smoke was 24.6 % (95 % CI: 22.3–27.0) for governmental employees, 29.9 % (95 % CI: 24.4–36.1) for non-governmental employees, 27.2 % (95 % CI: 21.5–33.7) for self-employed respondents, and 9.8 % (95 % CI: 8.2–11.7) for students. This percentage was 16.8 % (95 % CI: 15.6–18.1), 38.9 % (95 % CI: 32.9–45.2), 58.0 % (95 % CI: 39.7–74.4), and 60.7 % (95 % CI: 56.6–64.4) among those who never smoked, former smokers, current non-daily smokers, and current daily smokers, respectively.

## Discussion

Our findings, based on a large nationally representative study, showed high rates of smoking in Saudi men. Our study also revealed a high rate of daily smoking of shisha in both men and women. Moreover, we showed a high rate of exposure to smoking in the population. Our results show that smoking programs to prevent initiation of smoking and encourage quitting in those that do smoke should continue to be a priority for the Ministry of Health and other health partners and civil programs in KSA.

The 2005 STEPwise survey reported that the prevalence of smoking was 12.2 % (23.6 % for men and 1.5 % for females) among Saudis aged 25 to 64 years [11], compared to 15.3 % in our study (28.8 % for males and 1.9 % for females). However, STEPwise reported 25.9 % current smoking in men and 1.0 % for women 15 to 24 years old [11], compared to 16.1 and 0.8 % in our study, respectively. This decrease should be welcome news to the Saudi Ministry of Health that the current programs seem to be working for this age group and need to be strengthened and continued.

Shisha smoking increased from 3.34 % in STEPwise to 7.35 % in men in our study and from 0.5 to 1.28 % in women aged 15 to 64 years. In fact, our data show that shisha smoking increased among all age groups and both sexes. The trends reported in shisha smoking are of real concern. Unfortunately, shisha consumption is socially more acceptable than other kinds of smoking for women and for younger people in most countries of the Eastern Mediterranean region. [18, 19] This is compatible with our data that show very different male-to-female ratios for current smoking of all tobacco products and daily shisha smoking (19.6 vs. 5.6, respectively). There is a common misconception about the health effects of shisha, as many individuals believe that the water which is used in the instrument reduces the harmful effects of the product [18, 20]. The current trends in KSA and other countries of the region reveal that shisha is becoming more widespread and is commonly consumed in social gatherings, especially for the youth and younger adults.[21, 22] Our data show that strict measures should be developed and implemented to address the rise of shisha and that shisha smoking should be targeted more aggressively with appropriate prevention and cessation programs.

Several studies previously reported smoking status in KSA. Bassiony reported a range of prevalence of current smoking, from 2.4 % among female medical students to 52.3 % among primary care patients in a review article of 34 studies. [23, 24] Jarallah et al. reported a prevalence of current smoking of 21.1 % among men and 0.9 % among women (15 years or older) in three Saudi regions in 1990–1993. [8] Al-Nozha et al. (2009) reported current smoking prevalence of 12.8 % for 1995–2000 among individuals aged 30 to 70 years, which is a little lower than our estimate of 14.3 % for the same age group. However, their male-to-female ratio (2.6) was completely different from ours and the one reported by the STEPwise survey. [11, 25] The regional estimate by Al-Turki et al. for Al-Sharqia (16.9 % for 2004–2005) was also lower than our estimate (18.5 %) for the same region and age group. [9]

Our study has some limitations. First, our data are cross-sectional, and hence we cannot assess causality. Second, many of our behavioral data, such as smoking, are self-reported and subject to recall and social desirability biases. On the other hand, our study is based on a large sample size and used a standardized methodology for all its measures. Despite these limitations, our study remains nationally representative and has the merit of providing accurate data due to our near-real-time data quality monitoring through the whole survey period.

Men tend to smoke much more than women in most of the countries of the region. For example, current smoking is 38.1 % among men and 0.6 % among women in Egypt [26], compared to 23.4 and 1.4 % in Iran. [27] However, Turkey and Lebanon have different patterns with higher rates of smoking among women (15.2 % in Turkey and 29.8 % in Lebanon). [28, 29] It is crucial for the Saudi Ministry of Health to prevent increases in smoking among women. Historically, Saudi men were not heavy smokers (compared to the other Arab countries of the region) [29], but they picked up the trends of other neighboring countries. We hope that our findings will lead to ensuring that women in KSA do not follow the path of female smokers in Turkey or Lebanon. Although indicators of tobacco consumption in Saudi Arabia are better than for many other countries of the Middles Eastern region and high-income countries [30], there is potential for improvement in tobacco control.

KSA adopted the global FCTC in May 2005. [2] As a result, smoking is banned in public places such as governmental, educational, and health care facilities [3] However, we found high levels of exposure to secondhand smoke both among governmental and non-governmental employees and even those who work/study in educational institutions. The FCTC also requires prohibiting the sale of tobacco products to minors. However, some of the participants younger than 21 years of age reported smoking, indicating access to tobacco products and a lack of enforcement of the adopted legislations. [31] For KSA to succeed in reducing the burden of smoking, strong measures to enforce tobacco laws are needed.

The cigarette and cigar market in KSA is driven by multinational companies, mainly Philip Morris International and British American Tobacco. However, the shisha tobacco market is led by local and regional manufacturers such as Al Nakhla Tobacco Co SAE. [31] Tobacco products are reasonably cheap compared to the average income in KSA.[3] Perhaps it is time to increase taxation of tobacco products and use the revenues as additional sources for preventive efforts and enforcement. Moreover, stricter laws on local production and marketing should be imposed to curb the increasing trends in shisha smoking.

## Conclusions

Our study revealed that shisha smoking increased among all age groups, and smoking rates increased among those aged 25 to 64 years, despite the efforts of the Saudi Ministry of Health since signing and ratifying the FCTC treaty. Our findings call for the development and implementation of programs to prevent smoking initiation and encourage quitting. These programs are urgent, especially for women, as other countries in the region have reported much higher rates of smoking among females. KSA should also consider increasing taxation of tobacco products to achieve its health goals.
